# Effect of Nanoencapsulated Vitamin B1 Derivative on Inhibition of Both Mycelial Growth and Spore Germination of *Fusarium oxysporum* f. sp. *raphani*

**DOI:** 10.3390/ijms14024283

**Published:** 2013-02-21

**Authors:** Jeong Sub Cho, Yong Chang Seo, Tae Bin Yim, Hyeon Yong Lee

**Affiliations:** 1 DooSan EcoBizNet, Chuncheon 200-161, Korea; E-Mails: jschos@doosan.com (J.S.C.); taebini@doosan.com (T.B.Y.); 2 Department of Medical Biomaterials Engineering, Kangwon National University, Chuncheon 200-701, Korea; E-Mail: yongchang2da@kangwon.ac.kr; 3 Department of Teaics, Seowon University, Cheongju, Chungbuk 361-742, Korea

**Keywords:** thiamine dilauryl sulfate, nanoencapsulation, lecithin, spore germination, mycelial growth, *Fusarium oxysporum* f. sp. *raphani*

## Abstract

Nanoencapsulation of thiamine dilauryl sulfate (TDS), a vitamin B1 derivative, was proved to effectively inhibit the spore germination of *Fusarium oxysporum* f. sp. *raphani* (*F. oxysporum*), as well as mycelial growth. The average diameter of nanoparticles was measured as 136 nm by being encapsulated with an edible encapsulant, lecithin, whose encapsulation efficiency was about 55% in containing 200 ppm of TDS concentration: the 100 ppm TDS nanoparticle solution showed a mycelial growth inhibition rate of 59%. These results were about similar or even better than the cases of treating 100 ppm of dazomet, a positive antifungal control (64%). Moreover, kinetic analysis of inhibiting spore germination were estimated as 6.6% reduction of spore germination rates after 24 h treatment, which were 3.3% similar to the case of treating 100 ppm of a positive control (dazomet) for the same treatment time. It was also found that TDS itself could work as an antifungal agent by inhibiting both mycelial growth and spore germination, even though its efficacy was lower than those of nanoparticles. Nanoparticles especially played a more efficient role in limiting the spore germination, due to their easy penetration into hard cell membranes and long resident time on the surface of the spore shell walls. In this work, it was first demonstrated that the nanoparticle of TDS not a harmful chemical can control the growth of *F. oxysporum* by using a lower dosage than commercial herbicides, as well as the inhibiting mechanism of the TDS. However, field trials of the TDS nanoparticles encapsulated with lecithin should be further studied to be effectively used for field applications.

## 1. Introduction

White radish (*Raphanus sativus*) is an annual or biennial plant of the dicotyledonous papaverales crucifer family and is grown to get the large, fleshy roots. The edible part of the root has various shapes, including round, rectangular and long cylindrical, depending on the variant. It has existed widely in the temperate regions of China, South Korea, Japan and Europe [1]. One of the vegetables representing South Korea, white radish can now be grown throughout the year, thanks to the cultivation of new varieties with low thermosensitivity to low temperature and to the new subdivision of cropping patterns. The diseases and physiological disorders of white radish, however, are gradually increasing. Nineteen types of diseases in white radish have been reported to date, including Fusarium wilt and anthracnose [2].

One of the diseases of white radish, Fusarium wilt, first occurred in California, USA, and has spread all over the U.S. [3,4]. In South Korea, it was first reported in a white radish plantation in the suburbs of Chuncheon, and its incidence is gradually increasing, due to repeated cultivations [5,6]. *Fusarium oxysporum*, which causes Fusarium wilt in white radish, is a soil-borne Fungi imperfecti. It forms chlamydospores and exists in a dormant state in soil for several years, even with no host plant. Later, when the soil changes to an environment in which diseases occur, the chlamydospores germinate, enter the roots of the host plants and cause Fusarium wilt [7]. Various measures are being taken to control Fusarium wilt, such as crop rotation, soil fumigation, seed disinfection and prohibition of the overuse of nitrogen fertilizer, but these measures are not economical and have no obvious control effects. There is also no registered disinfectant for Fusarium wilt, making it difficult to control it [8]. Moreover, as most pesticides have the effect of controlling mycelial growth or killing plant disease pathogens, an efficient, reliable method for controlling spore germination is required to prevent Fusarium wilt, which starts with the germination of chlamydospores before the formation of mycelia.

Meanwhile, to prevent the spread of the aforementioned plant disease, many countries around the world, including South Korea, have recognized the need for eco-friendly biotic pesticides and are exerting much effort to develop related technologies and products. Most biotic pesticides are microbial pesticides, which are harmless to people, livestock and crops, while effectively controlling the target diseases and pests. Advanced countries have discovered beneficial microorganisms appropriate in their environments and have been utilizing these for many years. Global chemical and pesticide manufacturers have been focusing on the bioengineering projects of late and have been strengthening their strategy to advance to the biotic pesticide market. Particularly in South Korea, which has a relatively small land area and limited natural resources, there is an urgent need to maximize the national industrial competitiveness through the development of biotic pesticides that can actively respond to the global liberalization trend.

For this reason, the vitamin B1 derivative, thiamine dilauryl sulfate (TDS), which has been reported to have antifungal activity [9], was used in this study as a biotic pesticide. To efficiently enhance its effect of inhibiting the mycelial growth of plant disease pathogens, as well as its effect of controlling Fusarium wilt, which begins with the germination of chlamydospores, TDS was nanoencapsulated. This nanoencapsulation technology entraps the active components in the small size of 10^−9^ m, not only to lengthen their duration, but also to adjust their activation at the desired place. Thus, its use is gradually increasing [10,11]. In this study, the possibility of using TDS as a pesticide for inhibiting spore germination through the nanoencapsulation technology, to efficiently control the spore germination of *F. oxysporum*, which causes Fusarium wilt, was explored.

## 2. Results and Discussion

### 2.1. Production of TDS Nanoparticles and Identification of Their Characteristics

**Figure 1:**
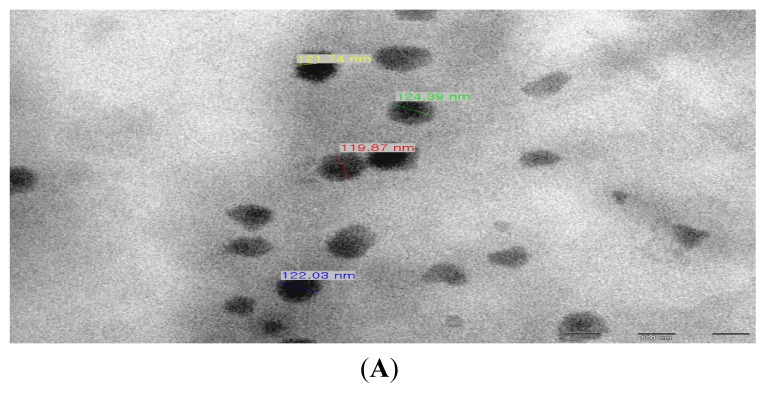

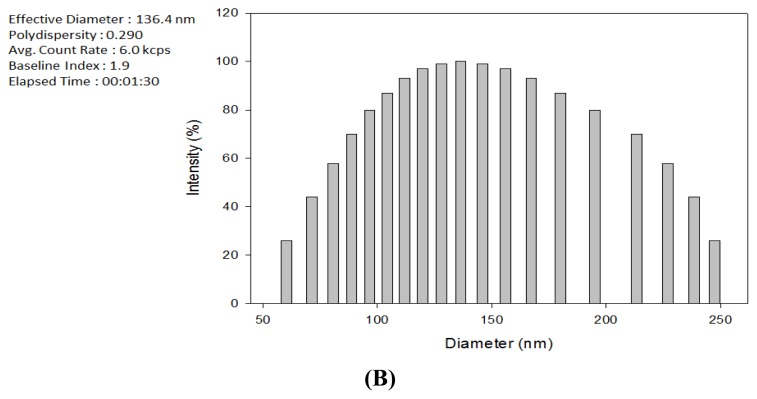
Characterization of the thiamine dilauryl sulfate (TDS) nanoparticle solution. (**A**) Photograph of the TDS nanoparticle solution from transmission electron microphotography (TEM); (**B**) Distribution of the TDS nanoparticle solution via dynamic light scattering (DLS).

Figure 1A shows the TEM photograph of the produced TDS nanoparticles that were 200 nm in size or smaller. It is shown that the nanoparticles were spherical and that they had a uniform size of around 120 nm. Their dispersibility in the solution phase was also excellent. Figure 1B shows the DLS measurement of the TDS nanoparticle solution. The mean particle size distribution of the TDS nanoparticle solution was 136 nm, which shows, along with the TEM photograph, that 150 nm or smaller nanoparticles were entrapped. This result suggests that entrapping TDS solution with edible lecithin makes it safe for use in food and can be directly applied to the manufacturing of spore germination inhibition pesticide. Furthermore, entrapping TDS solution in the nano size of 150 nm or smaller increases the surface area acting on the spores of *F. oxysporum*, as well as the spore penetration capability of TDS, thus generating a control effect greater than that of general pesticides acting on mycelia [9].

### 2.2. Encapsulation Efficiency of the TDS Nanoparticle

**Figure 2:**
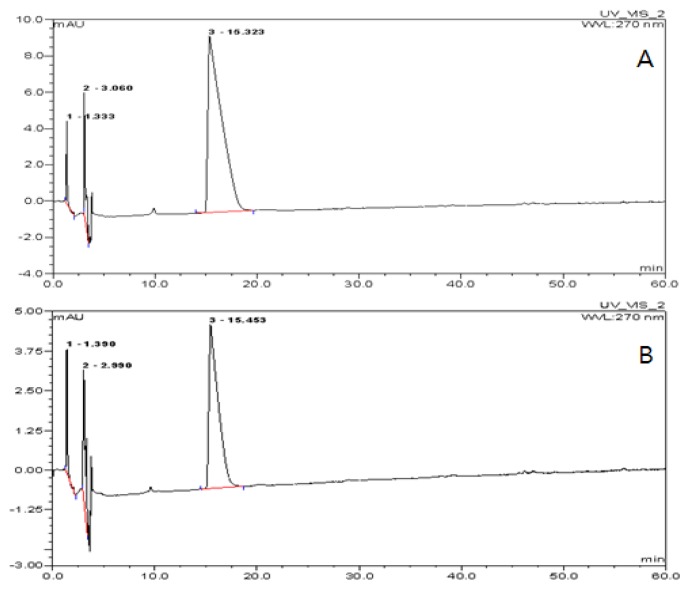
HPLC chromatograms of the TDS nanoparticle. (**A**) TDS standard, 100 ppm; (**B**) TDS after dissolving the nanoparticles.

To determine the encapsulation efficiency of TDS in the nanoparticles, TDS concentration in the solution was measured after dissolving the lecithin nanoparticles by HPLC analysis. Figure 2A was the TDS standard (100 ppm), and Figure 2B was the peak diagram of TDS buffer solution after being dissolved by PBS buffer by containing *ca.* 55 ppm of TDS. This result represented that the encapsulation efficiency of TDS nanoparticle with lecithin was about 55%, whose yield was relatively highly efficiency. This result was similar to the encapsulation efficiency of other water-soluble active substances in the ranges of 40%–60% [12]. Based on these results, it may be concluded that lecithin is a suitable carrier for the encapsulation of TDS solution.

### 2.3. Activation of the Inhibition of *Fusarium oxysporum* Mycelia Growth by the TDS Nanoparticle Solution

**Figure 3:**
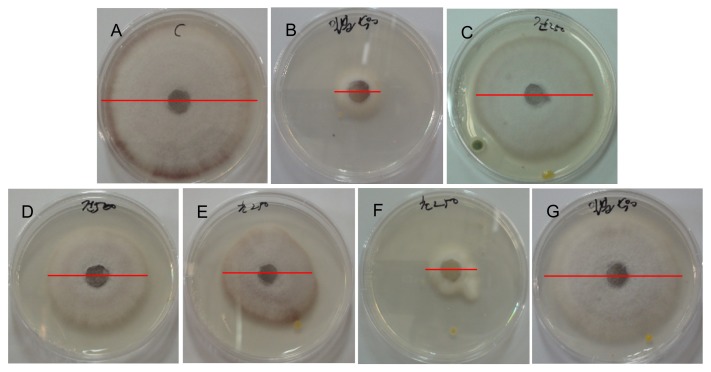
Comparison of the antifungal activities in the 20 mL TDS solution and 20 mL TDS nanoparticle solution for two different concentrations. (**A**) Negative control (no treatment); (**B**) positive control (dazomet), 100 ppm; (**C**) TDS solution, 50 ppm; (**D**) TDS solution, 100 ppm; (**E**) TDS nanoparticle solution, 50 ppm; (**F**) TDS nanoparticle solution, 100 ppm; (**G**) empty lecithin vesicles.

**Figure 4:**
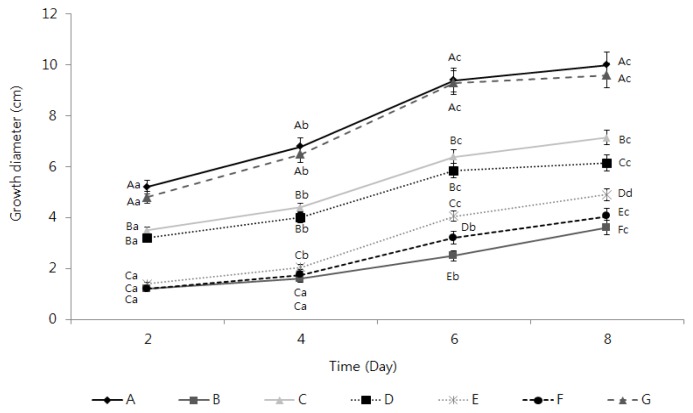
Growth of *Fusarium oxysporum* f. sp. *raphani* when different concentrations of TDS solution and TDS nanoparticle solution were added to it. (**A**) Negative control (no treatment); (**B**) positive control (dazomet), 100 ppm; (**C**) TDS solution, 50 ppm; (**D**) TDS solution, 100 ppm; (**E**) TDS nanoparticle solution, 50 ppm; (**F**) TDS nanoparticle solution, 100 ppm; (**G**) empty lecithin vesicles.

The observation of the inhibition of the mycelial growth of *F. oxysporum* by the TDS nanoparticle solution revealed that the mycelial growth inhibition effect appeared in every experimental group as opposed to the control group and that the growth inhibition effect increased along with the TDS concentration. The experimental group that showed the highest mycelial growth inhibition activity was that where 100 ppm TDS nanoparticle solution was used, whose efficacy was similar or slightly lower than the results in adding 100 ppm of dazomet, a positive control (Figure 3). Besides the data in Figure 3 that compared the inhibition efficacy at the end point of the experiments, kinetic analysis of inhibiting action of the samples were compared in Figure 4, according to the treatment time, whose result can better represent the action of the samples on the mycelial growth and spore germination. A mycelial growth inhibition rate of 38% was shown in adding 100 ppm of TDS itself, while the highest mycelial growth inhibition rate of 59% was shown in adding the same concentration of TDS nanoparticles, even though 64% of the highest inhibition was observed in adding a commercial herbicide, dazomet. For the case of adding lecithin (an encapsulant) itself, (**G**) in Figure 4, the mycelia growth was also almost similar to the case of no treatment (**A**). This result implied that the lecithin did not show a positive or negative effect on the mycelia growth for this experiment. These results suggest that using TDS nanoparticle solution can dramatically increase the surface areas acting on the mycelia of *F. oxysporum*, as well as the persistence of TDS in the mycelia, as opposed to using general TDS solution [13,14]. Moreover, whereas the water-dispersible powder of metalaxyl–copper oxychloride, a known chemical sterilizer, showed a 50% inhibition rate, the 100 ppm TDS nanoparticle solution showed a higher mycelial growth inhibition rate of 60% [15].

### 2.4. Measurement of the Spore Germination Inhibition Effect of the TDS Nanoparticle Solution

**Figure 5:**
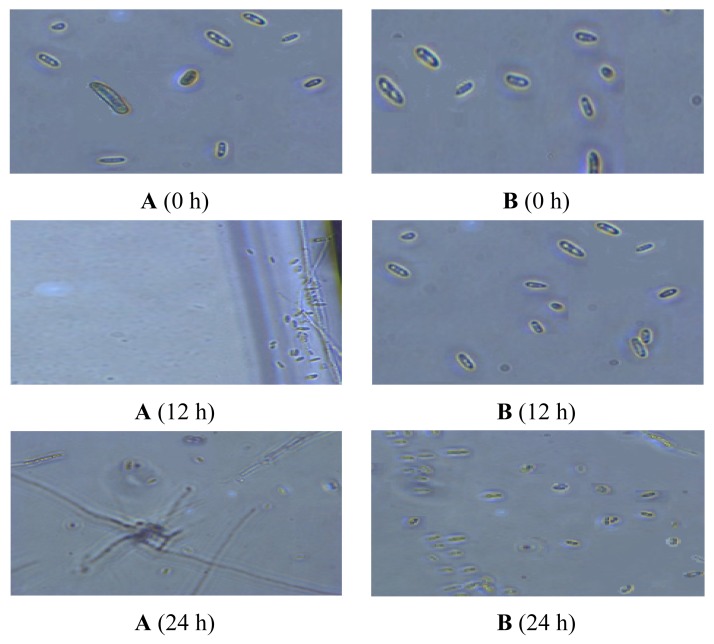
Spore inhibition in *Fusarium oxysporum* f. sp. *raphani* by TDS nanoparticle solution under microscope observation. (**A**) Negative control (no treatment); (**B**) TDS nanoparticle solution, 100 ppm.

To examine the spore germination inhibition effect of 100 ppm TDS nanoparticle solution, the spore germination was observed under an optical microscope at 0, 12 and 24 h (Figure 5). For the case of negative control (no treatment), spores were propagated to 1.29 × 10^3^ conidia/mL and fully germinated to the mycelia within 24 h after the inoculation, while the number of the spores was slowly increased only to 6.5 × 10^1^ conidia/mL and no visible growth of mycelia after 24 h in adding 100 ppm of TDS nanoparticles. This result implies that the TDS nanoparticle effectively inhibited the germination of chlamydospores and resulted in decreasing the mycelia growth.

**Figure 6:**
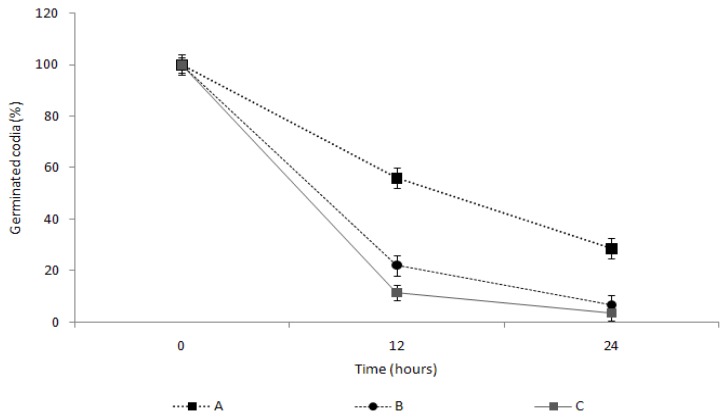
Effects of the TDS solution and TDS nanoparticle solution on *Fusarium oxysporum* f. sp. *raphani* spore germination. (**A**) TDS solution, 100 ppm; (**B**) TDS nanoparticle solution, 100 ppm; (**C**) positive control (dazomet), 100 ppm.

**Figure 7:**
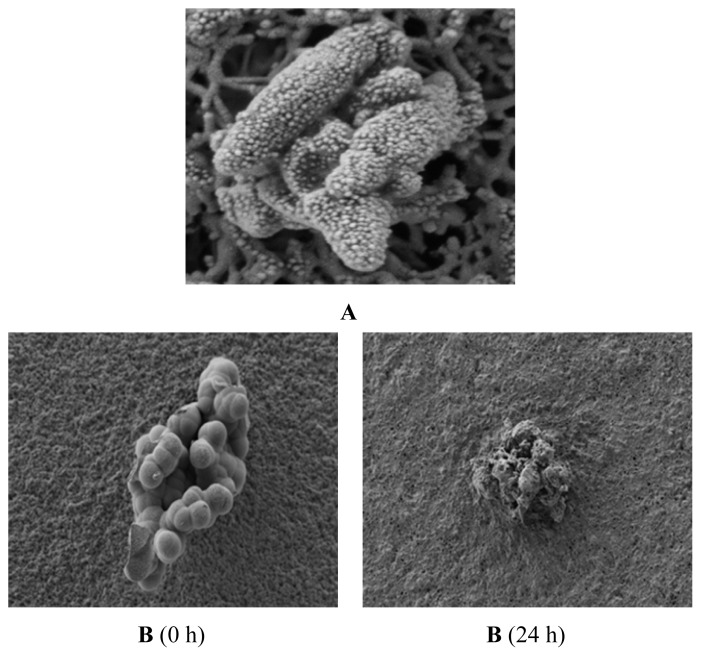
SEM images of the spores in *Fusarium oxysporum* f. sp. *raphani* after TDS nanoparticle solution treatment. (**A**) Negative control (no treatment) after 24 h incubation; (**B**) TDS nanoparticle solution, 100 ppm.

**Figure 8:**
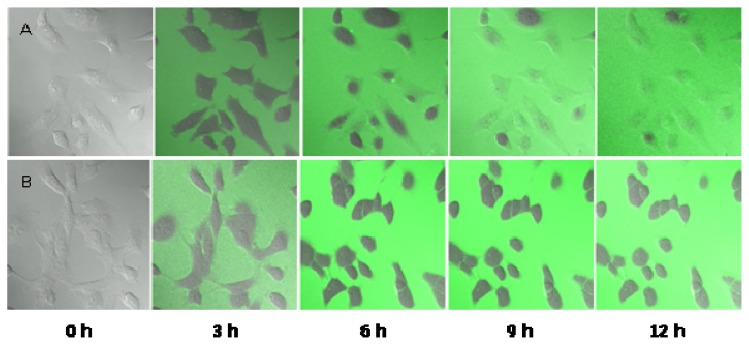
Confocal microscope images of the spores in *Fusarium oxysporum* f. sp. *raphani* in adding TDS nanoparticle solution, according to the treatment time. (**A**) TDS nanoparticle solution, 100 ppm; (**B**) TDS solution, 100 ppm.

To examine this spore germination inhibition effect in greater detail, the spore germination rate was measured by comparing with TDS itself and a commercial herbicide (Figure 6). The 100 ppm TDS solution showed the spore germination rates of 56.1% at 12 h and 28.6% at 24 h. In comparison, the 100 ppm TDS nanoparticle solution showed the spore germination rates of 22.3% at 12 h and greatly decreased down to 6.6% after 24 h, where the inhibition of the germination was very similar to the case of adding the dazomet (3.7% germination), even though for the first 12 h there was about a 15%–20% difference between the cases of TDS nanoparticle and dazomet. This result would suggest that TDS encapsulated nanoparticles required the time to start inhibiting the germination, compared to a herbicide, even though the nanoparticles can more effectively control the germination than TDS itself, possibly due to large surface areas and small sizes, enough to penetrate into the cell membrane and work in the cytosols [13]. This hypothesis can be proven by electron microscope observation of breaking the germs, as well as kinetic penetration of TDS nanoparticles into the germs, as shown in Figures 7 and 8.

### 2.5. Observation of the Spore Extinction Effect of the TDS Nanoparticle Solution

The spores were photographed under a scanning electron microscope (SEM) to observe the spore extinction mechanism of the TDS nanoparticle solution. Figure 7A shows the mycelia in the control group (without treating TDS nanoparticles), around which spores were well formed and even seemed to start germination. In comparison, Figure 7B shows that at 0 h, all of the spores in the disc looked like normal, but at 24 h, most of the spores were broken and started to be digested when adding 100 ppm of TDS nanoparticles.

To understand the inhibition mechanism of the TDS nanoparticle, the penetration of TDS nanoparticle into the germs was observed by confocal microscope, according to the treatment time in Figure 8. From the results, it was confirmed that TDS nanoparticles could penetrate into the spore membrane within 12 h and started to inhibit normal functions of the many kinds of molecules in the cytosol. After 12 h, FITC fluorescence started to be observed inside the spores and was evenly distributed throughout the spores after 6 h and completely covered the disc by breaking all of the germs. However, for the case of adding 100 ppm of TDS itself, the FITC labeled TDS was slowly passed through the cell membrane after 6 h, and the penetration was not very developed, even after 12 h treatment. This real-time confocal observation result first reported that the TDS nanoparticles can penetrate into the germs that are known to be very resistant and can work on inhibiting cell functions. It was suggested that cellular uptake of nanoparticles is more effective than uptake of micro-sized particles, owing to sustained-release properties, sub-cellular size and the target ability to a cell [16]. For a further detailed explanation of this observation, the effect of nanoparticle size on inhibiting the germination of the spores would be carried out, since most of the nanoparticle size was in the range of 150–200 nm in this experiment. A smaller particle size would be more effective in penetrating into the cell membrane, even though it would not be easy to make smaller sized nanoparticles. The tolerance of fungi towards chemicals may be due to changes in biochemistry of fungal cell walls that inhibit the entry of pesticides inside cells to a greater or lesser extent and, thereby, not reaching the site of action. Such changes may result in the decrease of the permeability of cell membrane and pesticidal detoxification, even before the site of action [17]. Conversion of a chemical in an active form may also be responsible for the detoxification mechanism [18].

## 3. Experimental Section

### 3.1. Cultivation of White Radish

Songbaek radish seeds were used for the experiment to establish the disease resistance test method. Horticultural bedsoil No. 5 (Bunongsa, Korea) was input into 8 × 16 linked pots (20 mL soil per pot, Bumnong, Korea), and the radish seeds were grown in the pots before they were cultivated in a greenhouse (25 ± 5 °C) for 14 days.

### 3.2. *Fusarium oxysporum* f. sp. *raphani Spores*

*Fusarium oxysporum* f. sp. *raphani* (KACC 40146, Korea) was used to investigate the spore germination inhibition activity. For the culture medium, potato dextrose agar (PDA, Sigma-aldrich, St. Louis, MO, USA) was used. The strains were cultivated at 25 °C for seven days, and the plectenchyma were removed from the colonies and were inoculated to malt extract broth (Becton, Dickinson and Co., Seoul, Korea). They were then shake-cultured at 150 rpm at 25 °C in a dark state for seven days. The cultivated strains were filtered through four layers of gauze to remove the hyphae, and the spore concentration was measured with a hemocytometer under an optical microscope (Zeiss Axioskop Microscope, Heidelberg, Germany). The samples were diluted with sterile water to the spore concentration of 1.0 × 10^−7^ conidia/mL.

### 3.3. Production of TDS Nanoparticle Solution

To produce the TDS solution that was used in this experiment, 100 g TDS powder was put in 1 L of 60% alcohol and was stirred for about 10 min, until it was completely dissolved. One milliliter of this TDS solution was added to 499 mL distilled water, and the resulting mixture was stirred for 60 min to produce 200 ppm TDS solution. To improve the safety of the crops, lecithin, which is edible, was used for the nanoencapsulation of the TDS solution. For lecithin, l-α-phosphatidylcholine (Sigma-aldrich, St. Louis, MO, USA) was used. It was melted in chloroform (Sigma-aldrich, St. Louis, MO, USA) and was put in a round bottom flask. All the chloroform was evaporated in a decompressed state to form lecithin multilayers. The TDS solution was added to the round bottom flask in which multiple lecithin layers were formed after complete drying and was homogenized at room temperature for one hour to produce TDS nanoparticle solution [19]. To collect 200 nm or smaller particles, it was filtered with a 0.2 μm syringe filter.

### 3.4. Observation of the TDS Nanoparticles

To check the particle shape of the produced TDS nanoparticles, the TDS nanoparticles were negative-stained with a phosphotungstic acid solution and were fixed with formvar/carbon. They were then thinly spread on a grid and dried. Then the nanoparticles were photographed under a transmission electron microscope (EF-TEM, LEO 912AB Omega, Carl Zeiss, Oberkochen, Germany) at 120 kV to capture their sizes and shapes [20]. To measure the uniformity and size distribution of the produced TDS nanoparticle solution, the size distribution of the TDS nanoparticles was measured via dynamic light scattering (DLS, Brookhaven Instruments Co., New York, NY, USA).

### 3.5. Measurement of Encapsulation Efficiency of the TDS Nanoparticle

The oversized nanoparticles in the solution were removed by gel-permeation chromatography using Sephadex G-100 columns (1.6 cm × 40 cm; bead size 40–120 μm) purchased from GE Healthcare (Uppsala, Sweden). The collected TDS nanoparticle fraction was centrifuged for 30 min at 16,770× *g*, and the precipitate was dissolved by adding 25 mL acetone (Sigma, St. Louis, MO, USA). After 30 min of stirring, 250 mg l-cysteine (Sigma, St. Louis, MO, USA) was added. The sample was sonicated at 60 kHz for 30 min and filtered through a 0.2 μm syringe filter [21]. The content of TDS in the filtrate was then determined by HPLC (M600E, M7725i/Waters, 996PDA, Waters, Milford, MA, USA). For HPLC analysis, a C18 column (250 × 4.6 mm, Waters, Milford, MA, USA) was used, where the mobile phase was water and acetonitrile and the gradient ratio was water:acetonitrile = 80:20 ~ 20:80 at 1.0 mL/min of flow rate and 270 nm absorbance. The injection volume was 25 μL.

### 3.6. Measurement of the *F. oxysporum* Mycelia Inhibition Effect of the TDS Nanoparticle Solution

The antifungal activity was measured to examine the *F. oxysporum* mycelia inhibition activity of the TDS nanoparticle solution. Strains of *Fusarium oxysporum* f. sp. *raphani* (KACC 40146, Korea) received from the National Institute of Agricultural Science and Technology were used for this experiment. Potato dextrose agar (PDA, Sigma-aldrich, St. Louis, MO, USA) was used as the culture medium, and the samples were cultivated at 20 °C in a dark state. For the measurement of the antifungal activity, a modified version of the method developed in [22] was used. For the experimental groups, general TDS solution and TDS nanoparticle solution were diluted to 50 and 100 ppm, respectively, and 20 mL volumes were used. A commercial herbicide, dazomet (3-5-dimethyltetrahydro-l-3-5-2 H thiadiazine 2 thione), was also used as a positive control. Each sample was added when the PDA culture media were produced. *F. oxysporum* strains were cultivated at 20 °C in a dark state for five days. A 0.5 cm colony was inoculated at the center of each of the media containing TDS solution and TDS nanoparticle solution, and the growth of the colonies was observed. To examine the growth inhibition rate in detail, the colony diameter was measured by date, and the mycelial growth inhibition rate was measured using the following formula. The experiment was conducted three times for each treatment group [9].

(1) $$\begin{array}{c} \text{mycelial growth}\;\text{inhibition rate }(\%)=(1-\text{diameter}\;\text{of mycelia in the TDS}-\text{treated}\\ \text{medium}/\text{diameter of mycelia in the no}-\text{treatment}\;\text{medium})\times 100 \end{array}$$

### 3.7. Measurement of the Spore Germination Inhibition Effect of the TDS Nanoparticle Solution

To examine the spore germination inhibition effect of the TDS nanoparticle solution, a PDA medium was produced with no TDS nanoparticle solution. For the experimental groups, TDS solution and TDS nanoparticle solution were added to the final concentration of 100 ppm during the production of the PDA medium, as well as the same concentration of 3-5-dimethyltetrahydro-l-3-5-2 H thiadiazine 2 thione (dazomet) as a positive control. For the control group and for all the experimental groups, spores with 1.0 × 10^3^ conidia/mL concentration were inoculated and cultivated at 25 °C in a dark state. After some time, the media on which the spores were cultivated were cut into 1 × 1 cm pieces and were observed under an optical microscope (Zeiss Axioskop Microscope, Oberkochen, Germany). To examine the spore growth inhibition rate, spores with 1.0 × 10^3^ conidia/mL concentration were inoculated to the media of the control and experimental groups. Then, they were cultivated at 25 °C in a dark state. The spore concentrations in the media cut into 2 × 2 cm pieces were measured over time. Furthermore, to examine the spore germination rate in detail, it was measured with the following formula using the spore concentration measured by hour. Three experiments were conducted for each treatment group.

(2) $$\begin{array}{c} \text{spore germination}\;\text{rate }(\%)=(\text{spore concentration in}\;\text{the sample}-\text{treated medium}/\text{spore}\\ \text{concentration in the no}-\text{treatment medium})\times 100 \end{array}$$

### 3.8. Observation of the Spore Extinction Effect of the TDS Nanoparticle Solution

To examine the mechanism of the TDS nanoparticle solution’s induction of spore extinction, the spores cultivated in the medium to which TDS nanoparticle solution was added during the measurement of the spore germination inhibition effect were observed. A scanning electron microscope was used to measure the surface. For the pretreatment, fresh samples were fixed in 2.5% glutaraldehyde solution at room temperature for 3 h, were washed with a 0.1 M phosphate buffer and were dehydrated with alcohol series. After dehydration, the samples were dried with a CO_2_ critical point drier (CPD2, Pelko, Melbourne, FL, USA), and the sample surfaces were gold-coated with a goldcoater (SC7640 sputter coater, Pocaron, Cambridge, UK) for 2 min. They were then observed with an electron microscope (LEO-435VP, SEM, North Billerica, MA, USA). Also, to image the penetration of TDS nanoparticles into the spore, a confocal laser scanning microscope (LSM510 META NLO, Carl Zeiss, Jena, Germany) was employed as follows: spores were prepared in the confocal dish; 200 μL of nanoparticles containing a fluorescein isothiocyanate (FITC) labeled TDS solution were then applied onto the spore and observed every 12 h until the germs were completely broken. The cross-section was imaged at 543 nm using a confocal laser scanning microscope [23].

### 3.9. Statistical Processing

The statistics of the experimental values were verified through paired *t*-test with the SPSS application. All the experimental values were expressed as mean ± standard error.

## 4. Conclusions

In this study, the vitamin B1 derivative thiamine dilauryl sulfate (TDS), which has been reported to have antifungal activity, was nanoencapsulated, and its effects in terms of the inhibition of the mycelial growth and spore germination of Fusarium wilt were investigated. Entrapping TDS solution with edible lecithin via nanoencapsulation makes the solution safe to use in food and allows it to be directly applied to the manufacturing of spore germination inhibition pesticide. Furthermore, entrapping TDS solution in the nano size of 150 nm or smaller increases the surface area acting on the spores of *F. oxysporum*, as well as the spore penetration capability of TDS, thus generating a control effect greater than that of general pesticides acting on mycelia [9]. This nanoencapsulation technology is used to entrap TDS, which has low stability in solution, as nano-sized particles and to increase its dispersibility in solution, thereby overcoming its low stability [24].

Using TDS nanoparticle solution increased the surface area acting on the mycelia of *F. oxysporum*, as well as easy penetration into the rigid germs [13], compared to TDS itself, to higher inhibition of mycelial growth and germination. Once TDS nanoparticles passed through the cell membranes of mycelia or spores, TDS can effectively inhibit the cell metabolisms, because being a vitamin B1 derivatives, TDS can strongly attach to the cell membranes and inhibit cell differentiation and proliferation in the cytosol [25]. According to this mechanism, the TDS nanoparticles inhibit cell division by attaching to the cell membranes of the mycelia or substantially inhibiting their proliferation by penetrating the protoplasm [26]. It showed greater mycelial growth inhibition activity than chemical pesticide, which is a water-dispersible powder of metalaxyl–copper oxychloride, a known chemical sterilizer. Entrapping the nanoparticles with edible lecithin improved the safety and decreased the residual toxicity, which is a problem of chemical pesticide, thus making it a safe biotic pesticide.

The measurement of the spore germination inhibition effect against *F. oxysporum*, which begins with the germination of chlamydospores before the formation of mycelia, showed the lowest spore germination rate in the TDS nanoparticle solution. The lecithin nanoparticles with hydrophobic surfaces were 100–300 nm in size and could easily penetrate the cells and facilitate the transportation of molecular matter, increasing the affinity to the cells [27]. Moreover, to improve TDS’s capability of penetrating the cells of soluble materials, many studies are being conducted to provide TDS with high penetration power and bio-utility by manufacturing active materials entrapped by oil as W/O-type liposomes [28]. The TDS nanoparticles in this study improved TDS’s persistence in the spores, its affinity and its spore penetration capability. The spore germination inhibition effect of the TDS nanoparticle solution showed a value similar to the reported spore germination inhibition effect value of hexanoic acid against *Botrytis cinerea* in tomato. Similar to the mechanism of acidifying the protoplasm to increase its cell membrane penetration capability, TDS was nanoencapsulated with lecithin, which has a hydrophobic surface and improved affinity to the cell membrane consisting of phosphatide, thereby improving the cell penetration capability of TDS [29]. Compared to the conventional pesticides inhibiting mycelial growth, it can control *F. oxysporum*, which begins with the germination of chlamydospores before the formation of mycelia, even with a low concentration. Furthermore, TDS nanoparticles can be used as an economic pesticide for the efficient control of spore germination.
